# Supplementary material for: Intestinal epithelial Dicer1 regulates gut microbiome and Alzheimer’s pathology in App-knock-in mice
Source: Alzheimers Res Ther. 2025 Sep 9;17:202. doi: 10.1186/s13195-025-01849-w (PMC12418706; doi:10.1186/s13195-025-01849-w)
Supplement: Supplementary file 1 — Supplementary Material 1 [file 13195_2025_1849_MOESM1_ESM.pdf]

Supplementary Table 1, Summarizing the number of animals in each experiment

			Number of mice	
		Genotype	male	female
Fig. 1	A - M	Dicer1-wt	2	2
		Dicer1-ko	3	3
	N	Dicer1-wt	5	3
		Dicer1-ko	3	3
	O - R	Dicer1-wt	6	3
		Dicer1-ko	3	3
Fig. 2	A - I	Dicer1-wt	2	3
		Dicer1-ko	2	7
Fig. 3	A	Dicer1-wt	3	4
		Dicer1-ko	2	3
	B - O	Dicer1-wt	4	3
		Dicer1-ko	3	3
Fig. 4	A and B	Dicer1-wt	5	7
		Dicer1-ko	6	5
	C	Dicer1-wt	3	2
		Dicer1-ko	3	1
	E, F, H, and I	Dicer1-wt	7	4
		Dicer1-ko	4	4
	D and G	Dicer1-wt	5	3
		Dicer1-ko	3	2
Fig. 5	A and B	Dicer1-wt	2	3
		Dicer1-ko	1	2
	C – E, and H	Dicer1-wt	3	5
		Dicer1-ko	3	3
	F and G	Dicer1-wt	3	4
		Dicer1-ko	3	3
	I and J	Dicer1-wt	1	5
		Dicer1-ko	1	3
	K and L	Dicer1-wt	5	3
		Dicer1-ko	2	2
Fig. 6	A and B	Dicer1-wt	3	5
		Dicer1-ko	2	3
	D - H	Dicer1-wt	2	5
		Dicer1-ko	2	3
Fig. 7	A and L	Dicer1-wt	7	5
		Dicer1-ko	4	3
	B - H	Dicer1-wt	4	4
		Dicer1-ko	2	4
	I - J	Dicer1-wt	4	2
		Dicer1-ko	2	2
	K	Dicer1-wt	4	4
		Dicer1-ko	2	2
Fig. 8	A - G	Dicer1-wt	6	3
		Dicer1-ko	3	3
	H - L	Dicer1-wt	4	8
		Dicer1-ko	4	5

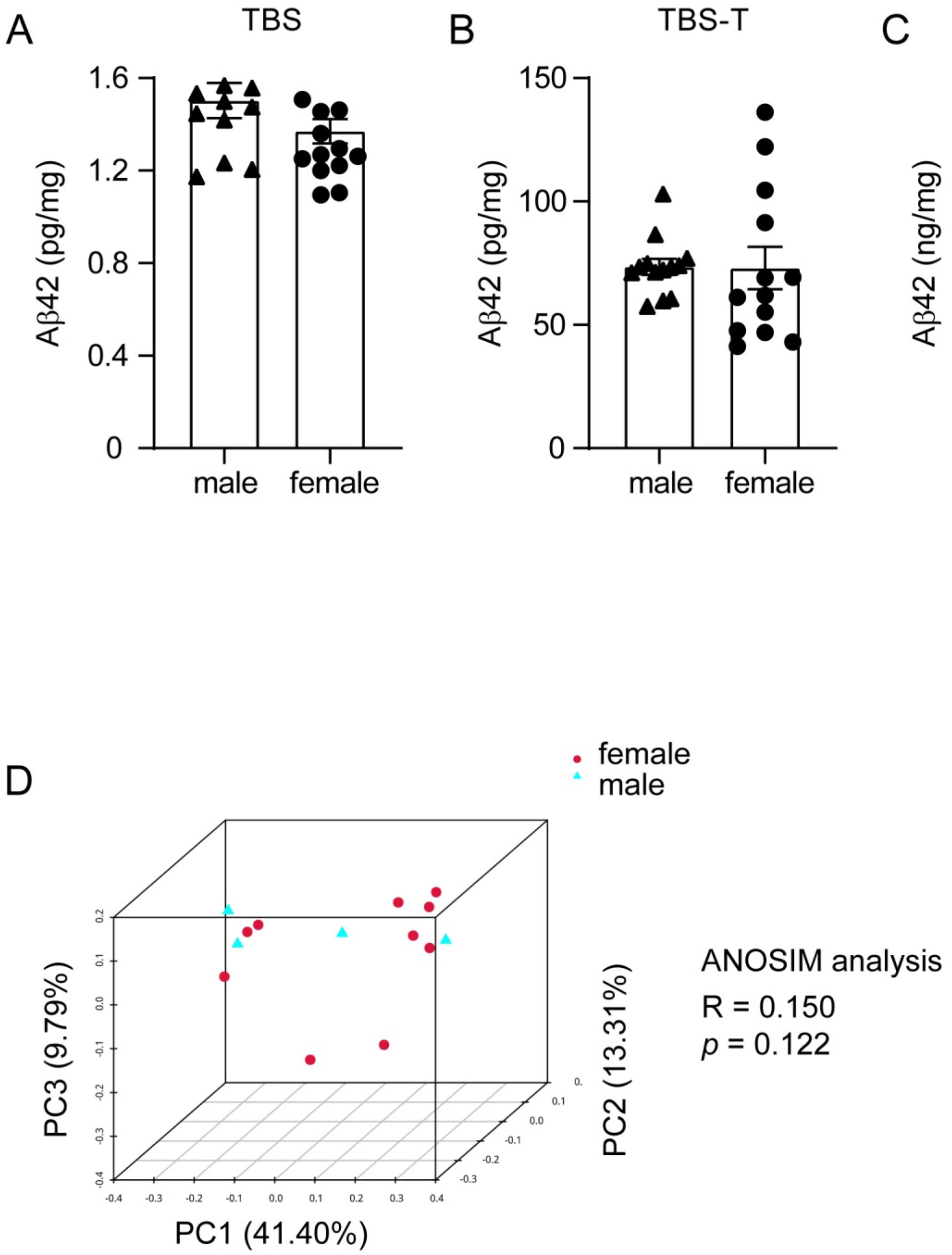

Supplementary Figure 1, Sex does not affect Aβ42 level in the brain and bacterial composition in the gut.

A – C: Cortex and hippocampus tissues were collected from 9-month-old male and female App(ki/ki) mice, serially homogenized in TBS, TBS plus 1% Triton-100 and guanidine-HCl for the extraction of Aβ, and then analyzed for Aβ42 levels using an ELISA kit. Aβ42 levels were normalized by the sample's protein concentration, which was determined by Bradford assay. Sex had no significant effect on cerebral Aβ42 concentrations in App(ki/ki) mice. t test, n = 12 and 15, for male and female mice, respectively.

D: Bacterial DNA was isolated from the cecum content of App(ki/ki) mice with and without deletion of Dicer1 in intestinal epithelial cells and sequenced for the V3-V4 region of 16S rDNA. Principal coordinate analysis (PCoA) was used for β-diversity analysis of bacterial composition at the genus level. ANOSIM analysis showed that sex does not alter the structure of intestinal bacterial community. n = 9 (male: 2) and 5 (male: 2) for AD mice with and without deletion of Dicer1, respectively.

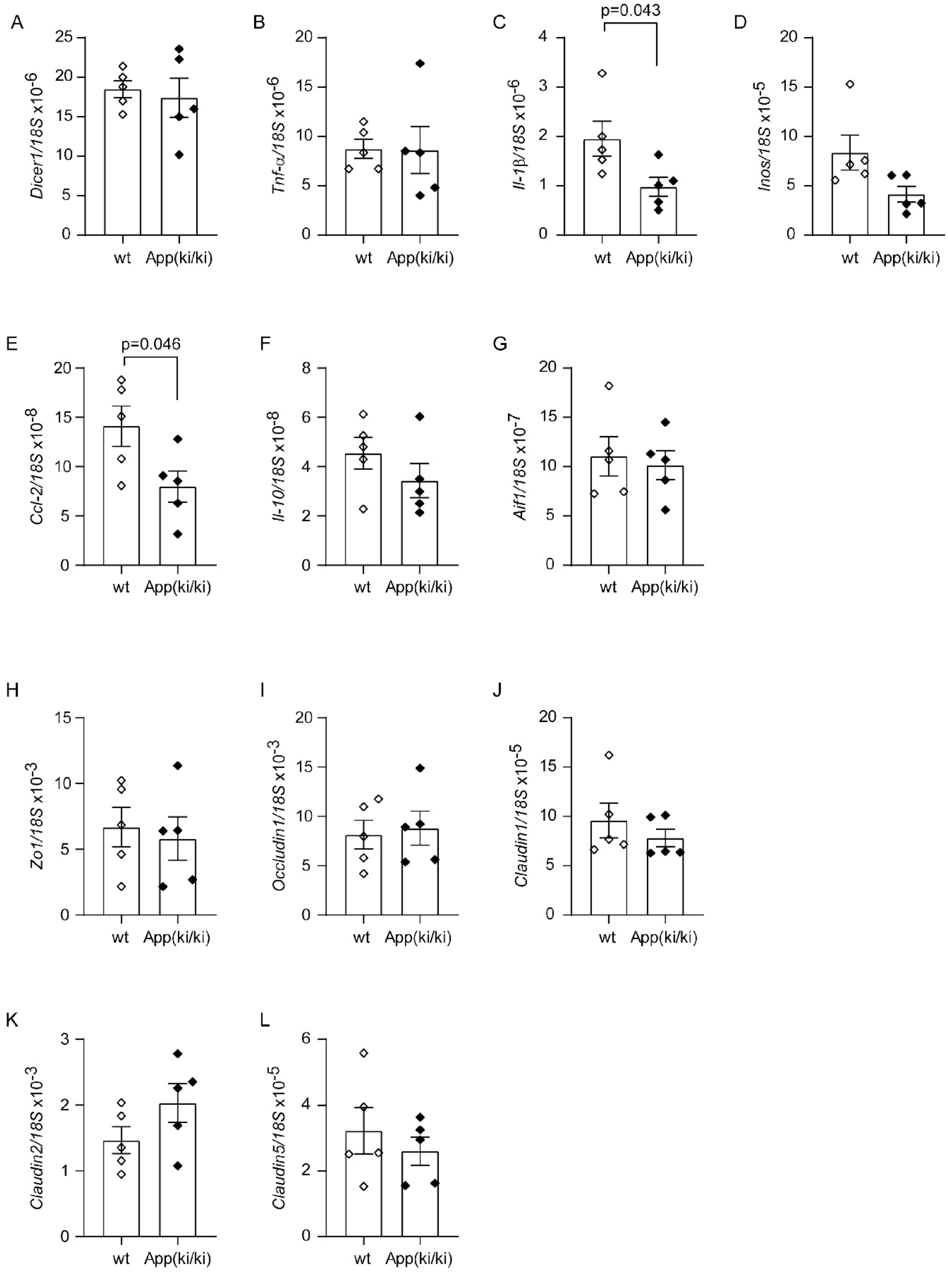

Supplementary Figure 2, Expression of AD-associated App mutant reduces inflammatory activation in the gut of mice.

Cortex and hippocampus tissues were collected from 9-month-old App(ki/ki) and App(wt/wt) littermate mice and analyzed for gene transcription by quantitative RT-PCR. Expression of humanized App containing AD-associated mutations reduced transcription of *Il-1β* and *Ccl-2* genes (C and E), but not other inflammatory genes tested (B, D, F and G), *Dicer1* gene (A) and TJP-encoding genes (H - L). t test, n = 5 (male: 3) and 5 (male: 4) for App(ki/ki) and App(wt/wt) mice, respectively.

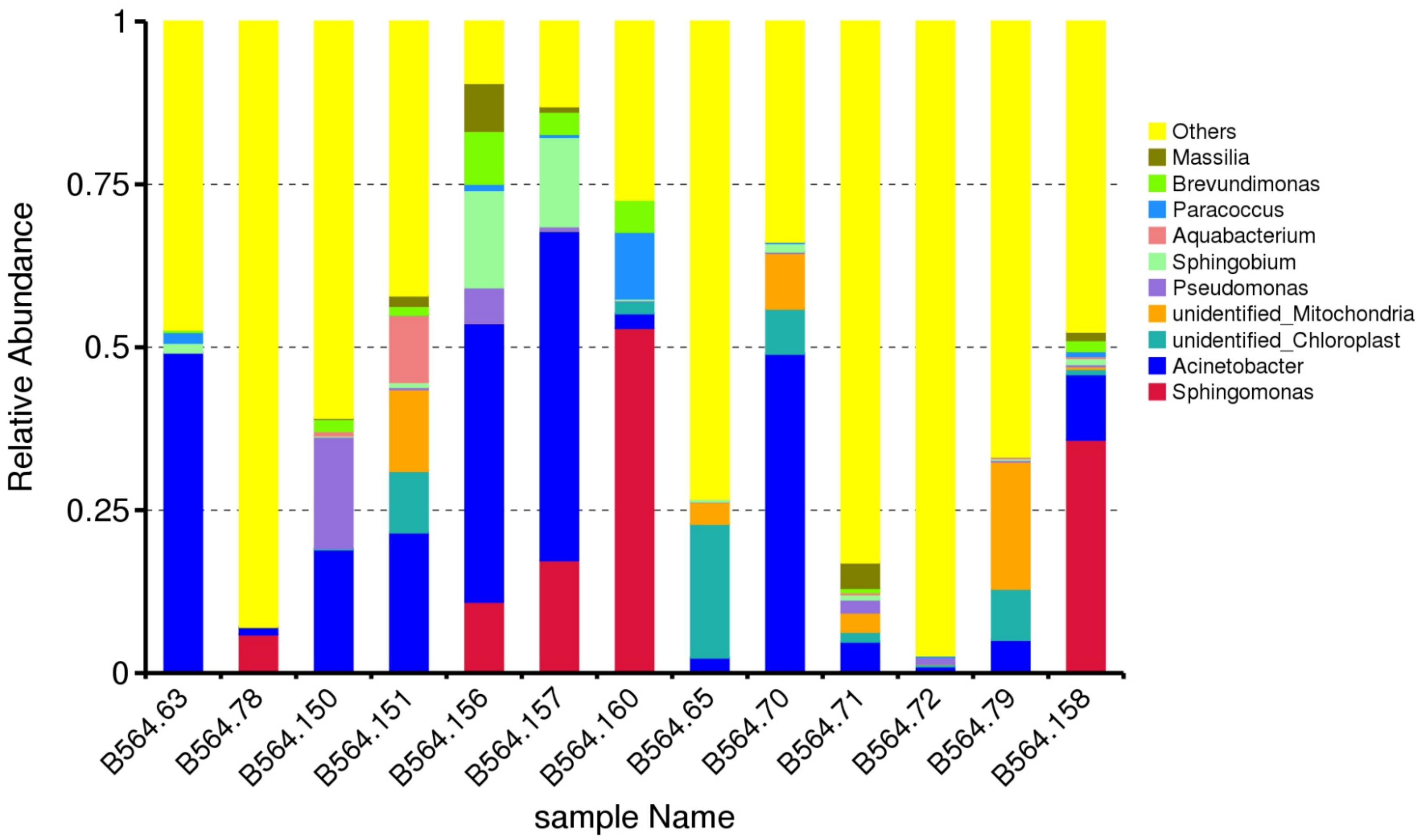

Supplementary Figure 3, Relative abundance of different bacterial genera in the brain tissues.

Total DNA was isolated from brain tissues of App(ki/ki) mice with and without deletion of Dicer1 in intestinal epithelial cells and sequenced for the V3-V4 region of 16S rDNA.

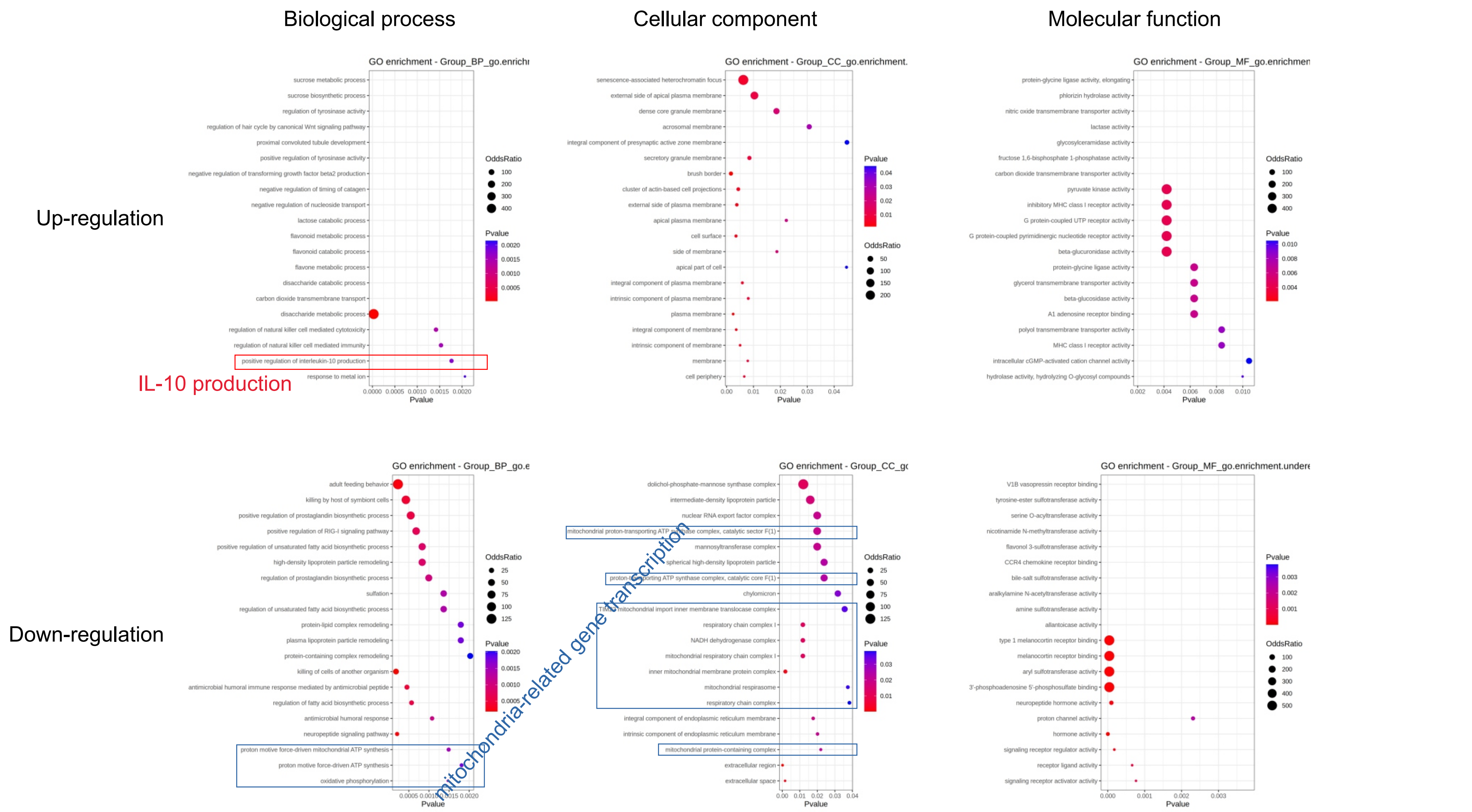

Supplementary Figure 4, GO enrichment analysis of differentially expressed genes.
Total RNA was isolated from brain tissue of App(ki/ki) mice with and without deletion of Dicer1 in intestinal epithelial cells and sequenced for mRNA. Differentially expressed genes (DEGs) were identified with a *p*-value (instead of adjusted *p*-value) cut-off at 0.05 and a minimum absolute log2fold change 1. GO enrichment analysis showed that these DEGs with upregulated transcription are linked to IL-10 production in Dicer1-deficient compared to Dicer1 wild-type App(ki/ki) mice, whereas the DEGs with downregulated transcription are enriched in mitochondria-related genes.
n = 7 and 12 for mice with and without deletion of dicer1, respectively.
