# Supplementary figures and images for: Intestinal epithelial Dicer1 regulates gut microbiome and Alzheimer’s pathology in App-knock-in mice
Source: Alzheimers Res Ther. 2025 Sep 9;17:202. doi: 10.1186/s13195-025-01849-w (PMC12418706; doi:10.1186/s13195-025-01849-w)

Raw images for Fig. 5, F

Raw images for Fig. 5, C

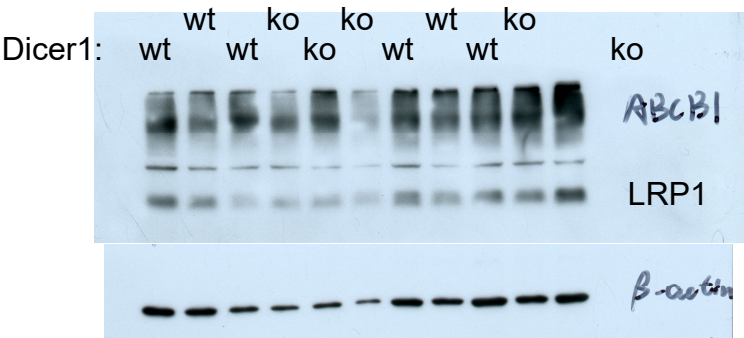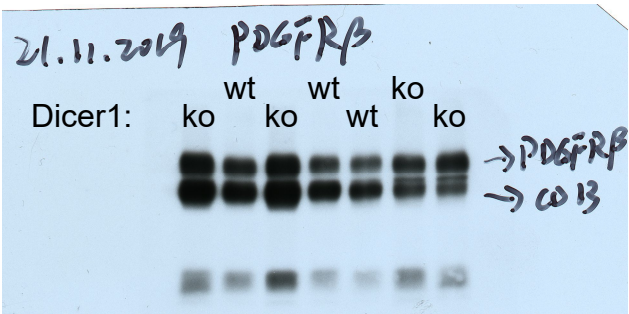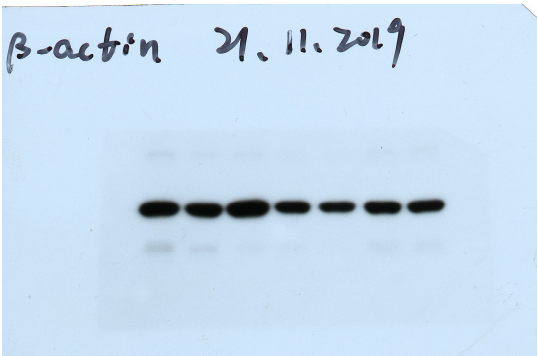

Raw images for Fig. 8, H

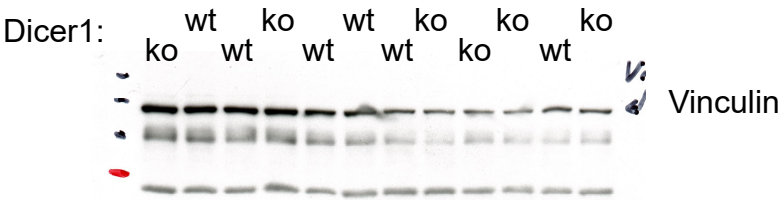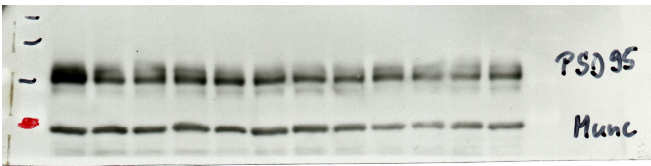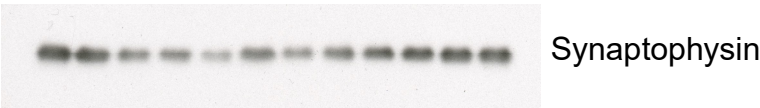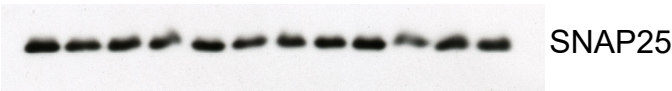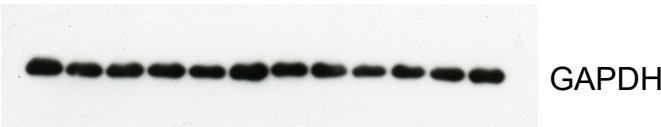

Supplement: Supplementary file 2 — Supplementary Material 2 [file 13195_2025_1849_MOESM2_ESM.pdf]
